# Ten-year changes in the psychosocial well-being, psychopathology, substance use, suicidality, bullying, and sense of coherence of 18-year-old males: a Finnish population-based time-trend study

**DOI:** 10.1007/s00787-020-01517-4

**Published:** 2020-03-30

**Authors:** Kim Kronström, Petteri Multimäki, Terja Ristkari, Kai Parkkola, Lauri Sillanmäki, Andre Sourander

**Affiliations:** 1 Department of Adolescent Psychiatry, Turku University Hospital, Hospital District of Southwest Finland, Turku, Finland; 2 Research Centre for Child Psychiatry, University of Turku, Turku, Finland; 3 Navy Command Finland, Turku, Finland; 4 Department of Child Psychiatry, Turku University Hospital, Hospital District of Southwest Finland, Turku, Finland

**Keywords:** Time-trend study, Adolescence, Mental health, Suicidal thoughts, Alcohol abuse, Drug abuse, Psychosocial well-being, Sense of coherence, Military call-up data

## Abstract

We studied Finnish 18-year-old males attending obligatory military call-up assessments in 1999 (*n* = 2340) and 2009 (*n* = 4309) on time-trend changes in psychosocial well-being, psychopathology, substance use, suicidality, bullying, and sense of coherence. Subjects filled in questionnaires, including the Young Adult Self-Report (YASR) for psychopathology and the Orientation to Life Questionnaire (SOC-13) for sense of coherence. The prevalence of minor mental health problems in the last 6 months decreased from 22.3% in 1999 to 18.6% in 2009 (OR 0.8, 95% CI 0.7–0.9), whereas severe mental health problems remained stable. Suicidal thoughts decreased from 5.7 to 3.7% (OR 0.6, 95% CI 0.5–0.8). The use of illicit drugs decreased from 6.0 to 4.7% (OR 0.8, 95% CI 0.6–0.95), but being drunk at least once a week increased from 10.3 to 13.4% (OR 1.3, 95% CI 1.0–1.5). Attention problems increased in YASR syndrome domains (mean score 2.9 vs 3.2, *p* < 0.001) and so did somatic complains (mean score 1.7 vs 1.9, *p* = 0.005). The SOC-13 scores remained stable. The percentage of males who had studied during the past 6 months increased from 91.4 to 93.4% (OR 1.3, 95% CI 1.1–1.6), while being employed decreased from 64.9 to 49.4% (OR 0.5, 95% CI 0.5–0.6). The positive findings included reductions in the prevalence of suicidal thoughts and the use of illicit drugs, but being drunk at least once a week increased. Self-reported somatic problems and attention problems increased. Despite changes in society and family structures, there were only minor overall changes in psychopathology.

## Background

There have been significant increases in the diagnosis and treatment of adolescent psychiatric disorders during the last 20 years. However, the evidence on whether this reflected changes in adolescent mental health at a population level has been inconsistent [1, 2]. A comprehensive review of secular trends in child and adolescent mental health by Collishaw [2] concluded that many studies found that adolescents’ emotional problems had shown a long-term increase over the past 30 years, especially among girls. Rates of antisocial behavior among adolescents appear to have leveled off, or fallen, since the 1990s in many countries. The use of services for several psychiatric disorders has increased and the reasons for this are probably partly related to broader diagnostic definitions and increased awareness and recognition by professionals [3–9]. Increases in mental health care and the use of psychotropic medication have been reported among children and adolescents [4, 6, 10–14].

There have been several population-based time-trend studies on changes in psychosocial well-being among adolescent males in Nordic countries. One Norwegian study reported that depressive symptoms among boys aged 16 to 17 more than doubled between 1992 and 2002, but did not continue to increase between 2002 and 2010. The same study reported that the level of conduct problems and daily smoking increased slightly and the use of cannabis more than doubled between 1992 and 2002. However, this was followed by positive trends in 2002 to 2010, when conduct problems and the use of both tobacco and cannabis more than halved [15]. A nationwide Finnish study that was based on a classroom survey and was carried out every second year from 2000–2001 to 2010–2011, showed a slight increase in self-reported depression among boys aged 14 to 16. The prevalence nearly doubled if their parents were unemployed and had low education levels [16]. Meanwhile, a study on changes in the self-reported mental health of Finnish boys aged 13 to 17 found fewer peer problems and better prosocial skills among boys in 2014 than in 1998. The study also showed a consistent decrease in smoking and alcohol use between the two time points [8, 17]. Overall, previous time-trend studies from the Nordic countries have showed trends towards better social skills and fewer conduct or behavioral problems among adolescent males. In the last 10 years or so, the stable or increasing trends in smoking, drugs or alcohol have started to decrease in many western countries. While several studies have showed that depressive symptoms have increased in girls over the last 20–30 years, the same trends have not been reported for boys [8, 15–17].

This study examined 10-year time-trend changes in psychological well-being among 18-year-old Finnish young men using confidential questionnaires completed during the military call-up process. Many changes and challenges take place in young men’s lives in late adolescence, including increased independence, and the psychological and social transition from adolescence to adulthood. It can be challenging for young men to pursue further education or find a foothold in the job market and, if they fail, they risk social exclusion. There were rapid changes in Finnish society, as well as in other western societies, between 1999 and 2009, including economic turbulence, increased technology, such as social media, and changing social circumstances [18, 19]. Many ongoing and overlapping temporal changes may have different effects on well-being. That is why is it essential to carry out methodologically sound epidemiological studies that provide information on trends in well-being. This information is important, as it helps us to develop both mental health services and social policy planning as a whole. However, one issue with these kinds of epidemiological studies are that when assessments are carried out at different time points they are not always based on directly comparable methods and the samples do not represent the whole population [2]. Our objective was to complement the existing information on trends by focusing on older adolescents, who have not been studied as much as other age groups. We were able to produce new information by using two questionnaires: the YASR [20], which focuses on pathology, and the SOC-13, which assesses sense of coherence [21, 22]. Using the same questionnaires in 1999 and 2009 meant that we were able to produce comparable and detailed data. A further strength was that we were able to provide good coverage of the study population, because gathering the information during the military call-up process meant that we reached the great majority of Finnish 18-year-old males. It is worth noting that in 1999 and 2009 Finland was one of the few European countries that still had compulsory military call-up for men at the age of 18. Women were able to join the military on a voluntary basis during these years, but the lack of a compulsory call-up meant that there was also a lack of data on women. That is why we focused solely on men. Participation in this study was on a voluntary basis and the results were confidential to encourage accurate self-reporting. Our previous study reported that the use of mental health services among 18-year-old men attending military call-ups was very low, at 3.2%. We also reported that there were no changes in the percentage treated between 1999 and 2009 [11].

The aim of this study was to report changes in a wide range of indicators, including well-being, living conditions, negative life events, substance use, peer relations, bullying, suicidality, sense of coherence and psychopathology. Our hypothesis, based on previous research with younger age groups from Finland, was that overall psychosocial well-being would be stable and we would see decreases in substance use and suicidality among Finnish 18-year-old males during the 10-year time period [8, 17].

## Methods

### Military call-up

The data for this study were gathered at military call-ups at two different time points, between September and November in both 1999 and 2009. Every Finnish male needs to attend call-up in the year that they become 18 years old. The purpose of the call-up system is to assess their suitability for military service. At both time points, the subjects were given the questionnaires during the call-up assessments and, if they decided they wanted to take part, they returned them in a sealed envelope to avoid reporting bias. The participants were reassured by the researchers that military personnel would have no access to the data that were collected.

### Study population

**Fig. 1:**
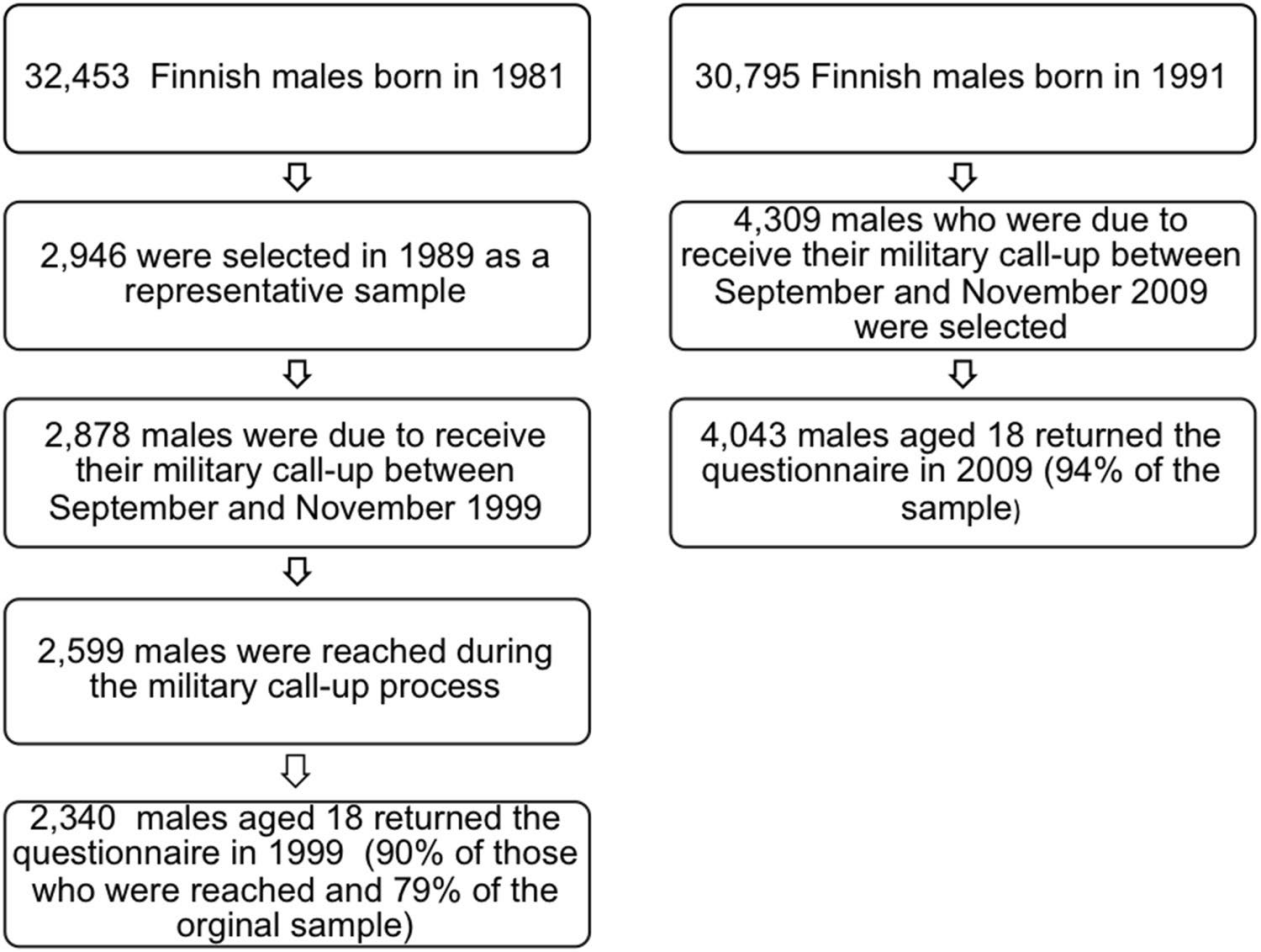
Flow chart on the 1999 and 2009 study populations

The 1999 sample was part of the nationwide From a Boy to a Man follow-up study, which was based on all 32,453 Finnish males born in 1981. In 1989, a 10% sample was drawn by selecting representative samples of communities according to their degree of urbanization: urban, suburban and rural. All the boys in small communities were included and representative subsamples were drawn from all school districts in larger cities. This identified 2946 boys, including 2878 who were due to receive their military call-up between September and November 1999. Of those, 2599 were reached and 2340 (90%) returned the questionnaires. The final responses represented 79% of the original sample (Fig. 1).

The 2009 study was based on 30,795 Finnish males born in 1991. To ensure that our random population-based sample was representative of our designated age cohort, we selected participants from four of the 19 geographical areas to ensure that they were representative of the country as a whole. However, the emphasis in this study was on the most densely populated southern parts of the country, as more young men lived in that region. We selected the areas covered by the Regional Offices in Varsinais-Suomi (south west), Helsinki (south), North Karelia (east) and Lapland (north). The target sample consisted of 4309 males aged 18 who were attending military call-up assessments between September and November 2009 in these four military call-up districts. A total of 4043 men (93.8%) returned the study questionnaires [11, 21].

### Questionnaires

At both time points, the subjects were asked to complete questionnaires that included questions about demographic factors, psychopathology, adaptive functioning, life events and risky behavior. The subjects were asked to provide answers that reflected their lives during the last 6 months.

It has been well established that family factors are determinants of health throughout life [23]. In order to study family changes, we asked the participants about three parental variables, namely divorce, death and severe illness. These were then dichotomized for analysis. They were also asked about where they lived, who they lived with, whether they had financial problems and if they had been studying or working in the last 6 months. We also asked them questions about their health and health-related behavior and categorized the responses as follows. Tobacco smoking was divided into no smoking, occasionally or up to five cigarettes a day or six or more cigarettes a day. Being drunk was never, less than once a week or at least once a week. Illicit drug use was never, had tried or currently used. Mental health problems were defined as emotional problems or problems related to concentration, behavior or getting along with other people and were divided into no problems, minor problems or serious problems. The duration of any mental health problems was less than a month, 1 to 5 months, 5 to 12 months or more than a year. Participants were asked if they had sought help for mental health problems during the past year or whether they had considered seeking help. The questionnaires also included questions on suicidal intentions and suicidal thoughts and whether the participant had been bullied or had been bullying others. The number of close friends was divided into none, one, two to three or four or more.

The psychopathology of the subjects was studied using the YASR [20], which has been found to be a valid instrument for assessing psychopathology in young adults [24]. The items are presented with three possible responses, where zero is not true, one is somewhat true and two is very true or often true. The YASR covers problems relevant to psychopathology, adaptive functioning, and social desirability. The present study used the 110 YASR items covering psychopathology [11, 20], which can be grouped together to form total problem scores and eight syndrome domains. The number of items per domain ranges from six for intrusive behavior to 16 for anxious/depressed. The domain on internalizing problems consist of 23 items and comprises withdrawn and anxious/depressed. Meanwhile, externalizing problems comprises 27 items and covers delinquent behavior, aggressive behavior and intrusive behavior. The other three items that were considered were somatic complaints, attention problems and thought problems. In 1999, we further analyzed the YASR syndrome domain distribution to establish the highest 10th percentile cut-off points for these scales to identify poor adaptive functioning. Using the same cut-of scores in 2009 enabled us to compare the number of young men who fell into highest 10th percentile of each scale in both study years.

Although there has been growing interest in researching adolescent resilience [26], the factors that relate resilience to stress are studied less frequently than the risk factors, assessment, and course of adolescent pathology. Sense of coherence arises from the salutogenic model of health [27] and reflects a person’s confidence and ability to have a meaningful and manageable understanding of their life and the environment they live in [28]. The Orientation to Life Questionnaire (SOC-13) was used to assess the sense of coherence [27, 28]. The scale consists of 13 items, which are rated between one and seven on a Likert-type scale and five of the items are reverse scored. The sum of all the items provides a score that ranges from 13 to 91. Higher scores indicate a stronger sense of coherence. Sense of coherence has been shown to be related to health, in terms of quality of life, health behavior, mental health, and family relationships [29].

### Statistical methods

Odds ratios (OR) with 95% confidence intervals (95% CI) were estimated for categorical outcomes using simple logistic regression analysis with the year as a predictor. The YASR domain sum scores were analyzed using analysis of variance with the year as the predictor. Two-sided p-values of less than 0.05 were regarded to be statistically significant. Homogeneity of variances was studied with Levene's Test. SAS version 9.4 for Windows (SAS Institute Inc, Cary, NC, USA) was used to conduct the statistical analyses.

## Results

The overall picture to emerge from our study of the self-reported psychosocial well-being of 18-year-old Finnish males was that there had been no major increases in most of the problem indicators when we compared the 1999 and 2009 cohorts.

### Living conditions

As seen in Table 1, the main change in where people lived from 1999 to 2009 was the increase in men living in cities with more than 100,000 inhabitants, from 17.6 to 22.9% (OR 1.4, 95% CI 1.2–1.6). Most of the subjects were still living with their parents, although living alone increased slightly from 4.8 to 7.0% (OR 1.5, 95% CI 1.2–1.9). There was an increase in the number of divorces in the subjects’ childhood families, from 30.3 to 34.8% (OR 1.2, 95% CI 1.1–1.4). Serious parental illnesses decreased from 7.9 to 2.1% (OR 0.2, 95% CI 0.2–0.3), as did paternal deaths, from 5.9 to 3.8% (OR 0.5, 95% CI 0.5–0.8).

**Table 1:** Background and psychosocial characteristics and substance use of participants in 1999 and 2009

	1999		2009		*p*	OR (95% CI)
	*N*	%	*N*	%		
Where males lived						
Countryside	467	20.3	757	19.0		Ref
< 10,000 inhabitants	432	18.8	825	20.7	0.049	1.2 (1.0–1.4)
10,000–50,000 inhabitants	777	33.8	1095	27.5	0.063	0.9 (0.8–1.0)
50,000–100,000 inhabitants	218	9.5	392	9.9	0.314	1.1 (0.9–1.4)
> 100,000 inhabitants	404	17.6	909	22.9	< 0.001	1.4 (1.2–1.6)
Who they lived with						
With parents	2097	90.0	3627	87.7		Ref
Alone	112	4.8	290	7.0	< 0.001	1.5 (1.2–1.9)
With a spouse	38	1.6	93	2.3	0.074	1.4 (1.0–2.1)
Other	82	3.5	128	3.1	0.507	0.9 (0.7–1.2)
If living with parents						
Both biological	1620	72.4	2592	66.2		Ref
Single parent	335	15.0	737	18.8	< 0.001	1.4 (1.2–1.6)
One biological and one step parent	171	7.6	370	9.5	0.002	1.4 (1.1–1.6)
Other	112	5.0	218	5.6	0.104	1.2 (1.0–1.5)
Divorce in childhood family	592	30.3	1408	34.8	< 0.001	1.2 (1.1–1.4)
Death of father	97	5.9	143	3.8	< 0.001	0.6 (0.5–0.8)
Death of mother	27	1.7	55	1.5	0.554	0.9 (0.5–1.4)
Parent´s serious illness	128	7.9	73	2.1	< 0.001	0.2 (0.2–0.3)
Studying during the past 6 months	2111	91.4	3831	93.4	0.003	1.3 (1.1–1.6)
Working during the past 6 months	1498	64.9	2032	49.4	< 0.001	0.5 (0.5–0.6)
Financial difficulties during past 6 months	240	11.0	300	9.4	0.050	0.8 (0.7–1.0)
Marriage or serious dating during past 6 months	900	39.0	1597	38.5	0.700	1.0 (0.9–1.1)
Tobacco smoking						
No	964	42.1	1743	43.9		Ref
Occasionally or 1–5 cigs a day	747	32.6	1100	27.7	< 0.001	0.8 (0.7–0.9)
6 or more cigarettes a day	580	25.3	1130	28.4	0.250	1.1 (0.9–1.2)
Being drunk						
Never	343	15.0	614	15.5		Ref
Less than once a week	1712	74.8	2818	71.1	0.257	0.9 (0.8–1.1)
At least once a week	235	10.3	529	13.4	0.027	1.3 (1.0–1.5)
Illicit drug use						
Have tried or used	138	6.0	184	4.7	0.018	0.8 (0.6–1.0)
Physical health problems						
Yes	416	18.0	814	19.7	0.100	1.2 (1.0–1.3)
Mental health						
No problems	1692	73.9	3205	77.4		Ref
Minor problems	511	22.3	769	18.6	< 0.001	0.8 (0.7–0.9)
Serious problems	88	3.8	168	4.1	0.954	1.0 (0.8–1.3)
Duration of mental health problems						
Less than a month	288	47.3	418	43.8		Ref
1–5 months	138	22.7	234	24.5	0.238	1.2 (0.9–1.5)
5–12 months	54	8.9	90	9.4	0.463	1.1 (0.8–1.7)
Over a year	129	21.2	213	22.3	0.341	1.1 (0.9–1.5)
Received help for mental health problems during past year						
No	2249	97.9	3973	97.2		Ref
Yes	48	2.1	113	2.8	0.100	1.3 (0.9–1.9)
Considered seeking help for mental health problems, but did not ask	44	2.0	116	2.9	0.034	1.5 (1.0–2.1)
Suicidal intentions	50	2.2	98	2.4	0.528	1.1 (0.8–1.6)
Suicidal thoughts	129	5.7	147	3.7	< 0.001	0.6 (0.5–0.8)
Being bullied	115	5.0	240	5.9	0.139	1.2 (1.0–1.6)
Bullying others	119	5.2	262	6.6	0.029	1.3 (1.0–1.6)
Number of close friends						
0	39	1.7	59	1.4		Ref
1	87	3.7	141	3.4	0.781	1.1 (0.7–1.7)
2–3	706	30.3	1163	28.0	0.688	1.1 (0.7–1.7)
4 or more	1495	64.3	2786	67.2	0.318	1.2 (0.8–1.9)
Feeling lonely	557	24.4	1024	25.4	0.351	1.1 (0.9–1.2)

Summary of separate logistic regression analysis results, with the study year as a predictor. *P* values and odds ratios (OR) with 95% confidence intervals (95% CI) are reported and Ref. refers to the reference category

### Studying and employment

The percentage of men who had studied or participated in training during the past six month increased from 91.4% in 1999 to 93.4% in 2009 (OR 1.3, 95% CI 1.1–1.6), while the subjects who had been employed during the same period decreased from 64.9 to 49.4% (OR 0.5, 95% CI 0.5–0.6).

### Tobacco, alcohol, illicit drugs

The percentage who did not smoke tobacco and those who smoked six or more cigarettes a day increased between 1999 and 2009, from 42.1 to 43.9% and from 25.3 to 28.4%, respectively, but the changes were not significant. At the same time, those who smoked occasionally or up to five cigarettes a day decreased from 32.6 to 27.7% (OR 0.8, 95% CI 0.7–0.9).

The number of men who had never been drunk was 15.0% in 1999 and 15.5% in 2009 There was a slight trend towards heavier alcohol use, as the percentage of men who reported being drunk at least once a week increased from 10.3 to 13.4% over the study period (OR 1.3, 95% CI 1.0–1.5). Illicit drug use decreased from 6.0 to 4.7% (OR 0.8, 95% CI 0.6–1.0).

### Physical and mental health

The number of subjects who reported no current physical health problems remained stable at more than 80% in 1999 and 2009. A single question was used to cover mental health problems, which were defined as problems with emotions, concentration, behavior or getting along with other people. The prevalence of males who reported that they had experienced minor mental health problems in the last 6 months decreased significantly, from 22.3% in 1999 to 18.6% in 2009 (OR 0.8, 95% CI 0.7–0.9), whereas severe mental health problems remained stable (3.8% vs 4.1%).

### Suicidality

There were no changes in reported suicidal intentions from 1999 to 2009 (2.2% vs 2.4%). At the same time, the prevalence of subjects who reported suicidal thoughts decreased from 5.7 to 3.7% (OR 0.6, 95% CI 0.5–0.8).

### Bullying, friends, and loneliness

There were no changes in the prevalence of males who reported that they were bullied from 1999 to 2009 (5.0% vs 5.9%). However, the percentage of subjects who reported bullying others increased from 5.2 to 6.6% during the same period (OR 1.3, 95% CI 1.0–1.6). No changes were found in the percentage of males who reported feeling lonely (24.4% vs 25.4) in both years. In both years, less than 2% reported that they had no close friends (1.7% vs 1.4%).

### The YARS questionnaire

**Fig. 2:**
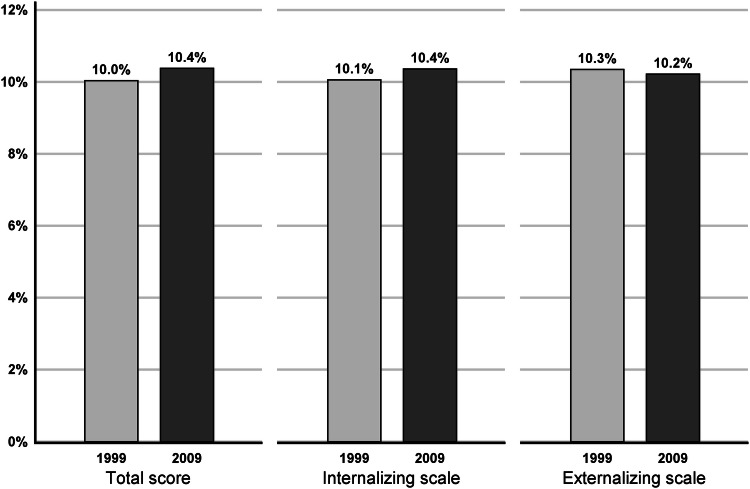
The percentage of participants in the highest 10th percentile, using the same cut-off point in 1999 and 2009 and the total YASR scores, internalizing and externalizing scale scores of participants in 1999 and 2009. No significant changes were found

Psychopathology and adaptive functioning were assessed with the YARS questionnaire. As seen in Table 2, there were no significant changes in the mean values of the total, internalizing or externalizing scale scores between 1999 and 2009. In the YASR syndrome domains there was an increase in attention problems (mean score 2.9 vs 3.2, *p* < 0.001) and somatic complains (means score 1.7 vs 1.9, *p* = 0.005). There were no significant changes in the other YASR syndrome domains: anxious/depressed, withdrawn, thought problems, intrusive, aggressive behavior and delinquent behavior. The distribution of the YASR syndrome domain scores was studied by analyzing the percentage of participants in the highest 10^th^ percentile using the same cut-off point for 1999 and 2009. This showed that the only statistically significant change was the increase in subjects with high scores for attention problems, from 7.1% in 1999 to 8.7% in 2009 (OR 1.24, 95% CI 1.02–1.51, *p* = 0.03). No changes were found in the percentage of subjects in the highest 10^th^ percentile for the total YASR score or the internalizing and externalizing scale scores between 1999 and 2009 (Fig. 2).

**Table 2:** YASR domain scores of participants in 1999 and 2009

	1999			2009			*p*
	*N*	Mean	SD	*N*	Mean	SD	
YARS scores							
Total score	2282	23.7	17.8	3999	24.3	17.8	0.194
Internalizing scale	2277	6.3	6.0	3919	6.5	6.0	0.238
Externalizing scale	2281	6.1	5.3	3925	5.8	5.3	0.110
YASR syndrome domains							
Anxious/depressed	2283	4.2	4.3	3960	4.3	4.4	0.464
Withdrawn	2278	2.1	2.2	3956	2.2	2.2	0.074
Somatic complaints	2273	1.7	2.2	4010	1.9	2.3	0.005
Thought problems	2277	0.4	0.9	3965	0.4	0.9	0.448
Attention problems	2275	2.9	2.2	3963	3.2	2.2	< 0.001
Intrusive behaviour	2282	2.0	1.9	3931	1.9	2.0	0.076
Aggressive behavior	2284	2.8	2.6	3976	2.8	2.6	0.571
Delinquent behavior	2284	1.2	1.9	3963	1.1	1.8	0.113
SOC-13	2295	66.9	10.8	4106	66.7	10.8	0.440

Mean values and standard deviations (SD)

*p* value of pairwise year comparisons

### The SOC-13 questionnaire

**Fig. 3:**
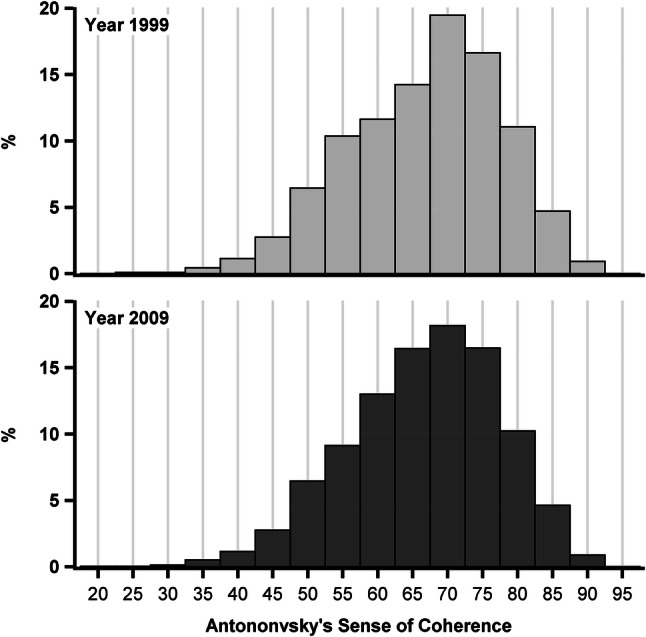
The distribution of SOC-13 sum scores (range 13 to 91) in 1999 and 2009. No significant changes were found

The SOC-13 was used to assess the sense of coherence and we found no differences in the mean values between 1999 and 2009. No statistical significance was found in the difference in variances between 1999 and 2009 (Levene’s Test *p* = 0.67). The distribution of the SOC-13 scores was almost identical in both years (Fig. 3).

## Discussion

The overall picture that emerged from this study was that the psychosocial well-being and mental health of Finnish males called up for military service in 1999 and 2009 either stabilized or showed minor improvements. The six-month prevalence of 18-year-old men who reported having no mental health problems, no suicidal thoughts and no illicit drug use slightly increased. In contrast to these positive findings, the number of young men who reported getting drunk at least once a week increased. Despite changes in society and family environments, psychopathology—as measured with the YASR—remained very stable, as did sense of coherence, which indicates resilience.

Ongoing changes in society and family structures were reflected in the results, including the fact that it became more common to live in larger cities and alone from 1999 to 2009. Both urbanization [30, 31] and living alone [32] may pose mental health challenges, particularly for vulnerable individuals. There were more parental divorces in 2009 than in 1999. Previous studies showed that many children experienced distressing thoughts and emotions when their parents were getting divorced and divorce was a risk factor for mental health problems among offspring [33]. However, another study reported that most subjects did not experience serious outcomes [34]. Research has shown that when family structures changed, good emotional connections between adolescents, their parents and other family members were protective against poor health outcomes [23, 35]. In accordance with rising life expectancy [36], there were fewer serious parental illnesses and paternal deaths reported in 2009.

Between 1999 and 2007 there was strong economic growth in Finland, which was reflected by just 9.4% of the men in the 2009 cohort reporting financial difficulties. However, the global economic turmoil from 2007 onwards had a strong negative effect on job markets in Finland [36], which could be a major explanation for the significant decrease in working men found in 2009. In 1999, more than two-thirds of the men had worked during the last 6 months, whereas in 2009 this had fallen to just half. It is worth noting that military call-ups took place during the autumn months, so the employment rate included summer jobs. Adolescents often see working as a way to earn extra money rather than the opportunity to learn new skills [37]. Despite this, work experience seems to play an important role in career identity development [38]. At the same time, the number of men who had been studying during the last 6 months increased slightly from the already high rate of 91.4% in 1999 to 93.4% in 2009. Access to education has been shown to be one of the strongest determinants of health among adolescents worldwide [23] and the fact that a high percentage of our two cohorts had been studying was considered to be a protective factor against marginalization. It is possible that the diverse trends in working and studying found in this study cannot be explained solely by the changes in opportunities to work or study. The decrease in the percentage of adolescent men working from 1999 to 2009 may also have been partly due to changes in the aims and values of the adolescents. Employment opportunities were more limited in 2009, due to the global recession, and young men could have felt that it was better to focus on their education, rather than search for a job, so that they could improve their position in future labor markets. Based on previous studies, there seems to be a trend towards work being less important and leisure time being more highly valued [39]. As most of the men who responded were studying and financially better off, their motivation to seek jobs may have declined from 1999 to 2009.

More than 80% of the men reported no current physical health problems in both years. The reported six-month prevalence of perceived minor mental health problems decreased from 22.3 to 18.6%, while perceived severe mental health problems remained stable, at about 4.0%. A similar trend was reported for suicidality: the less severe form of suicidality decreased, namely suicidal thoughts, while more severe suicidality, defined as suicidal intensions, remained stable. The few previously published population-based studies on recent trends in suicidal thoughts among adolescent boys are consistent with our study. Suicidal thoughts among male American high school students decreased between 1991 to 2011, while more severe suicidality, namely suicide attempts, remained stable [40]. Another American study found no changes in either suicidal ideation or suicidal attempts between 1995 and 2005 among boys in late adolescence [41]. Likewise, a Dutch study reported no changes in the prevalence of suicidal ideation between 1993 and 2003 [42]. To conclude, these population-based studies showed stability or decreases in the prevalence of suicidal thoughts and stability when it came to suicidal ideation or attempts by adolescent males.

It has been shown that high rates of self-reported problems in childhood and adolescence are strong predictors for psychiatric diagnoses in adulthood [43]. The YASR internalizing scale has also been shown to effectively identify present diagnosed depression or anxiety, as defined in the Diagnostic and Statistical Manual of Mental Disorders, 4th Edition [45]. Several previous time-trend studies have showed increases in depression and anxiety disorders among girls, but did not report the same results for mental health among boys [8, 15–17, 43, 46]. Our study showed that the YASR internalizing scores were stable and this suggests that there were no major trends in depression from 1999 to 2009 among 18-year-old Finnish men. This was in line with previous studies on younger boys. However, we did find a slight increase in the YARS syndrome scale for somatic complains.

An increasing trend in self-reported somatic complains was reported among eight-year-old Finnish boys between 1989 and 2005 [46] and Norwegian boys aged 11–16 between 1994 and 2014 [47]. In our previous study, somatic complains among 18-year-old males showed an independent correlation with several psychiatric disorders during the five-year follow-up period [48]. It is possible that the association between somatic complains and psychiatric symptoms is at least partly mediated by the stress the individuals were facing [49–53].

Our study suggests that there was a small, but significant, increase in reported attention problems among males during the 10-year time period, as both the mean scores and the prevalence of males with high YASR syndrome scale scores for attention problems increased. Growing concerns have been expressed that Internet use and gaming could lead to increases in attention problems [54]. However, we are not aware of any previous evidence of time-trend increase in the prevalence of attention problems when comparable evaluation methods were used. Studies of eight-year-old Finnish children [55] and boys in mid-adolescence [8] did not find any increases in attention problems. It is possible that increased public discussions have led to growing awareness of attention problems and attention deficit hyperactivity disorder (ADHD) among 18-year-old males and that more cases have been identified. It is worth noting that ADHD medication was almost non-existent in Finland in the 1990s and has increased rapidly since early 2000 [56–58]. It is likely that the use of ADHD medication became more common among men attending the military call-up during the 10-year period of our study. It is also possible, that using such medication would have had effects on reported attention problems and affected our results. Previous studies have not reported any actual increases in attention problems, but diagnoses of ADHD have substantially increased, which appears to be due to increased awareness and changes in diagnostic practice [2, 3].

Sense of coherence, as an indicator for resilience, has previously been shown to be associated with lower substance use, better social skills [59] and life satisfaction [60] and to predict how adolescents react to stress in different cultures [61]. Our previous study showed that poor sense of coherence scores at the age of 18 were independently associated with anxiety, depression, antisocial personality, and substance use disorder during the five-year follow-up period, when they were controlled for the effect of psychopathology at baseline [48]. As far we know, there have not been any previous population-based studies on trends of sense of coherence among adolescents. Our study found an almost identical distribution of SOC-13 scores in 1999 and 2009. The lives of 18-year-old men changed during those 10 years, such as the increase in social media use [62]. However, it is notable that their sense of coherence, defined as their confidence and orientation with regard to their lives and environments, remained unchanged.

Bullying is an important risk factor for adolescent health. Bullying victimization has been associated with later mental health problems, such as anxiety [63–66], depression [63–67], and suicidality [65]. Furthermore, bullying has been associated with somatic health problems and various psychosocial problems and socioeconomic disadvantages [65, 66]. Previous time-trend studies on bullying prevalence have reported decreasing trends [10, 68–71] or stable trends [34]. However, these studies covered younger age groups than our study, which focused on 18-year-old males. In our study, there was no change in the prevalence of young men who reported that they had been bullied, while those who admitted bullying others increased slightly from 1999 to 2009 (5.2 vs 6.6%). The reported prevalence of both bullying and being bullied were very low compared to the results of studies on younger age groups. It is likely that this was partly due to the fact that bullying has been reported to decline with age [72].

Excessive alcohol use and smoking are major public health concerns. Heavy alcohol use in late adolescent is a strong predictor of excessive alcohol use and alcohol-related problems in adulthood, [73, 74]. Initiatives to decrease the consumption of alcohol and cigarettes include legislative acts, such as advertising restrictions, tax on cigarettes and alcohol, bans on public consumption and minimum legal ages for purchasing and using products [75]. Although campaigns to prevent substance use are common, the evidence on their effectiveness is still very low [76, 77]. Furthermore, alcohol policy changes do not seem to influence alcohol consumption [78] and legally restricting the sale of tobacco to minors has not been associated with changes in adolescents’ smoking rates [79]. Many previous studies [33, 71, 80–83] have reported decreasing trends in smoking and alcohol consumption among adolescents. In line with these studies, our research found a decrease in occasional tobacco smoking. However, there was a rather minor increase, from 10.3 to 13.4%, in the prevalence of young men who reported being drunk at least once a week. Although it has been reported that reductions in alcohol use may not be as strong in adolescent males as females [17], and among 18-year-olds than younger age groups [18], no comparable study has reported an increase in heavy drinking on a weekly basis. This group of young men who reported being drunk every week are at risk for excessive alcohol use and alcohol-related problems in later adulthood [73, 74]. However, occasional drunkenness, which was reported by over two-thirds of our subjects, has been reported to be normal in men of this age [84].

### Strengths and limitations

The strength of the study was that it was representative of the target population and used the same standardized instruments to measure psychopathology at both time points. The 1999 study collected national data from Finnish men attending mandatory call-up assessments in the year they turned 18. The 2009 study data were also collected during the military call-up process and we aimed to ensure that it was geographically representative and comparable to the 1999 study population. The participation rate in the 1999 sample was 90% and it was 94% for the 2009 sample. Because of the large number of subjects, and the time-trend approach, the self-report rating scales provided useful information. However, they lacked the specificity that formally structured interviews may have provided. As the participants were asked to reflect on the previous 6 months, recall bias may have affected the reports. It is important to emphasize that the present findings, which showed limited changes in self-reported psychical and mental health, were restricted to Finland and males. These trends may have been different if we had studied adolescent girls, because recent evidence has suggested that girls´ emotional problems have increased [2, 8].

## Conclusion

This study was carried out at an important stage in the development of our subjects, as it focused on 18-year-old males who were attending mandatory on-call military assessments and undergoing the transition from adolescence to adulthood. Psychopathology and psychosocial disadvantages during this phase of life increase the risk of a wide range of adversities, including social exclusion [85]. Therefore, population-based time-trend studies that examine possible changes in psychosocial well-being are very important when planning interventions. This is because they can help policy makers and other stakeholders to understand how changing environments affect adolescent well-being. We found stability or minor improvements in the overall psychosocial well-being of the males. Self-reported somatic complaints and attention problems increased during the follow-up period. A novel finding that did not emerge from previous time-trend studies was that a stable sense of coherence indicated resilience during the 10-year follow-up period. The positive findings included reductions in the percentage of young men reporting mental health problems or suicidal thoughts. The 30% increase in the prevalence of young men reporting heavy alcohol use is a worrying finding. Changes in society were reflected in the financial situations and work experiences of the young men, but the levels of pathology and resilience remained stable.
